# Comparison of Whey Versus Almond Protein Powder on Nitrogen Balance in Female College Students; The California Almond Protein Powder Project (CAlmond-P^3^)

**DOI:** 10.3390/ijerph182211939

**Published:** 2021-11-13

**Authors:** Adeline Maykish, Morgan M. Nishisaka, Courtney K. Talbott, Scott K. Reaves, Aleksandra S. Kristo, Angelos K. Sikalidis

**Affiliations:** 1 Department of Food Science and Nutrition, California Polytechnic State University, San Luis Obispo, CA 93407, USA; amaykish@calpoly.edu (A.M.); mnishisa@calpoly.edu (M.M.N.); cktalbot@calpoly.edu (C.K.T.); sreaves@calpoly.edu (S.K.R.); akristo@calpoly.edu (A.S.K.); 2 Cal Poly Personalized Nutrition Research Group, California Polytechnic State University, San Luis Obispo, CA 93407, USA

**Keywords:** muscle protein synthesis (MPS), nitrogen balance (NB), protein powder, protein supplementation, nutritional analysis, hydration, urine specific gravity (USG)

## Abstract

Plant-based diets have become increasingly popular in the past decade, with approximately 11% of Americans self-identifying as vegan or vegetarian and many others trying to reduce meat consumption. Due to increasing interest, the plant-based food market has significantly expanded, with several innovative products serving as alternatives to animal-based products. One such example is almond protein powder, a new protein supplement created as an alternative to whey protein. Due to the novelty of almond protein products, little is known regarding how well the protein supplement supports nitrogen metabolism. The effects of both an almond-based protein beverage and a whey-based protein beverage on nitrogen balance are investigated in the work presented herein. Twenty female college students aged 20–25 years were randomly assigned to consume either an almond- or whey-based protein drink twice daily for one week; 24-h urine collection was performed at the baseline and endpoint of the 7-day treatment period and nitrogen balance was assessed. Body composition and hydration status were also assessed. Both protein sources (almond and whey) were able to notably improve nitrogen balance, thus indicating that almond protein powder may be a functional plant-based alternative to whey protein powder and may be of interest in future research regarding muscle mass and body composition improvement.

## 1. Introduction

Current Western diets consist of considerable amounts of highly processed foods, red meats, and high-fat dairy products, with a significant portion of dietary protein from animal-based protein sources [1,2]. Meat production requires substantial land use, with 30 million square kilometers worldwide, accounting for 60% of the world’s agricultural land use [3]. Beef production results in a fossil-fuel-to-energy ratio of 40:1, and all animal-based proteins demonstrate an average ratio of 25:1. This ratio is 11 times greater than that of grains, thereby illustrating a significant impact of animal-based protein consumption on the environment [4]. Greenhouse gas emissions vary across protein sources as well, with beef producing 60 kg CO_2_ equivalents/kg, whereas soy produces less than 1 kg of CO_2_ equivalents, as plant-based proteins typically favor sustainability more than animal-based [5,6].

While plant-based food alternatives extend several environmental benefits, it is critical that they function in a similar manner, nutritionally, to animal-based protein sources to encourage consumers to modify dietary habits without sacrificing nutritional benefits. Plant-based protein functionality has been compared to that which is animal-based on several occasions [7,8,9], with evidence suggesting decreased disease risk and numerous health benefits with plant-based options [8]. However, plant-based proteins have exhibited mixed effects on muscle protein synthesis (MPS) in human studies when compared to animal-based protein, suggesting that for those who are prioritizing muscle-building maximization, animal-based proteins may still constitute the best option [8,9].

Almond protein powder (produced from almond; *Prunus amygdalus*), a more recently commercialized protein powder, has not been thoroughly investigated in terms of its effects on MPS. There is evidence suggesting that soy and pea protein powders may not be as effective as whey protein due to their differing amino acid profiles. The amino acid profile of almond protein powder differs from other plant-based proteins, consumed in vegan or vegetarian diets. Specifically, almond protein powder has a different essential amino acid profile compared to both soy and pea protein [10]. This may result in different effects on MPS. Investigating the functionality of almond protein powder compared to a well-established animal-based counterpart is important for developing nutritional recommendations for vegetarians/vegans interested in consuming more protein and ensuring an adequate amount and quality of dietary protein intake.

In this context, we performed a proof-of-concept study in which the first objective was to determine the body composition in relation to protein status of female college students, including lean body mass, percentage body fat, and visceral fat mass. It was hypothesized that female students would be marginal in terms of their protein intake or display lower levels of protein consumption compared to nutritional guidelines [11].

The second objective of our study, presented herein, was to assess body composition and nitrogen balance during a protein supplementation period and compare the effects from using either an animal- or plant-based protein source. It was hypothesized that both supplements would improve the participants’ marginal protein status, as evaluated through nitrogen balance assessment.

The third and final objective was to compare the effects of the protein source, either animal- or plant-based, on nitrogen balance and protein status. It was hypothesized that there would be a similar positive effect on nitrogen balance, regardless of the supplementation source.

## 2. Materials and Methods

### 2.1. Participants

Twenty female full-time college students aged 20–25 years were recruited for this pilot study. Participants were eligible if they were within an age range of 20–25 years, lacked allergies to dairy or tree nuts, were able to consume both animal and plant protein, had no present chronic diseases, did not regularly consume protein supplements (3× or more per week), and did not take medication for hypertension, glycemic control, or dyslipidemia, or antibiotics. Participants were informed of the purpose of the study, the experimental procedures to be used, and any potential risks. All participants were not pregnant at the beginning of the study. Written consent via an informed consent form was obtained from all participants before beginning the study. The experimental protocol and all procedures followed were approved by the Human Subjects Research Committee of California Polytechnic State University, San Luis Obispo (IRB No. 2021-091), prior to the commencement of the study.

### 2.2. Experimental Protocol

The experimental protocol implemented was designed to examine the effect of receiving whey versus almond protein supplementation on nitrogen balance. A randomized single-blind design was used, whereby study participants were assigned to either the whey (n = 10) or almond (n = 10) supplementation group through randomization in Excel. In addition, individuals completed 3-day dietary records and exercise logs prior to supplementation to assess nutrient differences. At the initial and post-supplementation test days, participants’ anthropometry was performed, including BMI, height (cm), weight (kg) (EB9380H Bodyweight Scale, Etekcity), percentage body fat and muscle mass, and percent body water. Body composition, bone density, fat mass, and muscle mass were assessed using dual-energy X-ray absorptiometry (DXA) at baseline. Secondary analysis of body fat, muscle mass, water, basal metabolic rate (BMR), and visceral fat was assessed using bioelectrical impedance. Participants performed 24-h urine collections at baseline and at the conclusion of the study. 24-h urea nitrogen and urine specific gravity were analyzed to determine effects on nitrogen balance (NB) and hydration using standard protocols.

### 2.3. Protein Supplementation

Protein supplementation was provided in the form of protein powder containing commercially available whey (animal source) or almond (plant source) protein powder. Powders were then added to unsweetened almond milk and enriched chocolate flavoring to create a protein shake. Both shakes were the same in protein amount, color, taste, and texture. Ingestion of the supplement was twice daily over the course of seven days and provided 30 g (15 g each serving) of additional protein per day to each participant. Individuals were asked to maintain their typical dietary and exercise habits throughout the experimental period (1 week). The almond shake contents consisted of 34.5 g of almond protein powder (providing 15 g of protein), 16.8 g of vitamin-enriched chocolate flavoring, and 295 mL of unsweetened almond milk per drink. Whey shakes consisted of 17 g of whey protein powder (providing 15 g of protein), 16.8 g of vitamin-enriched chocolate flavoring, and 295 mL of almond unsweetened milk per drink. This totaled 15 g of protein added per shake (whey protein powder contains a more concentrated amount of protein than almond, thus requiring less powder of the former to reach equal protein amounts per intake). Unsweetened almond milk was used in both the whey and almond protein shakes to ensure uniformity. The total amount of protein ingested per day from the diet and the provided supplementation combined was within safety guidelines, as available from the American Society for Nutrition, with recommendations not to exceed 175 g/day (35% energy from protein in a 2000 kcal diet) for a 64 kg (140 lb.) person [12]. Caloric content for each serving of the shakes included 285 kcal/shake for the almond shake and 205 kcal/shake for the whey shake. Participants were provided all the ingredients on campus during the first meeting, along with written instructions, and were asked to make their own drinks in their place of residence due to concerns pertaining to COVID-19. Supplement and shake ingredients were provided to each participant in individually packaged containers and included clear containers with lids and a plastic scoop (Table 1). Shaker bottles for drink production were also provided. Compliance with the supplementation protocol was monitored via the distribution of a twice-daily survey through e-mail.

### 2.4. Dietary Analyses and Consultations

Dietary intake was recorded in the 72 h prior to supplementation. Standardized dietary assessment procedures were utilized throughout the study. Participants used standardized 3-day food and exercise records created by the Sports Nutrition Team at California Polytechnic State University, San Luis Obispo [13], to record what they consumed three days prior to supplementation. All dietary records included two weekdays and one weekend day. Time of day, portion size, food description (including brand), and preparation method were all recorded. Participants were given a portion size guide to estimate food amounts. Exercise logs consisted of date and time of day, duration, and exercise description. Records were then entered into ESHA Food Processor Nutrition Analysis software (ESHA Cloud Services, Version 1.5) to obtain values regarding the nutritional intakes of participants, including daily calorie and macronutrient intake, and their relation to the recommended values. ESHA’s default recommended values are based on the participants’ profiles, including age, sex, height, weight, and activity level. All participants were determined to be “lightly active”, unless no exercise was reported on the exercise log and they were, thus, determined to be “sedentary”. Additionally, participants were asked to consistently consume the same dietary intake as recorded in their three-day food records during the week-long protein supplementation phase.

### 2.5. Body Composition

Body composition was assessed at the Nutrition and Health Assessment Laboratory in the Food Science and Nutrition department at California Polytechnic State University, San Luis Obispo. Full body scans were performed using DXA on a Lunar iDXA (GE Healthcare, Madison, WI, USA), and all procedures were completed according to GE Lunar specifications. Weight, height, and ages were recorded immediately before the DXA scans. Bone density, fat mass, and fat-free mass were assessed using DXA data at baseline; 12-h fasting was required prior to scanning, and all participants were required to present a negative pregnancy test before the scan. DXA baseline analysis allowed the comparison of participants’ data to extensive databases as a means of confirmation that the studied population was healthy and within normal body composition parameters (DXA data not shown). Bioelectrical impedance analysis (BIA) was also performed via a Tanita MC-780U Plus P (Tanita Corp., Tokyo, Japan). BIA was conducted both at baseline and at the conclusion of the study. Participants stood barefoot on the metal sole plates, and a 1 kg tare was entered as per the manufacturer’s recommendation to account for light clothing. All measurements were performed using standard prediction equations as no participants were determined to require the athlete setting. BIA was utilized to determine total body fat, muscle mass, visceral fat, body water, and BMR. Moreover, both methods for body composition assessment (BIA and DXA) were utilized at baseline to indicate that the groups (all participants) were not different as per body composition at baseline.

### 2.6. Nitrogen Analysis

For nitrogen analysis, 24-h urine collection was performed at baseline and at the conclusion of the study. Participants consumed protein shakes for 7–8 days, including on the last day they completed their urine collection. Participants delivered their own urine samples to Pacific Diagnostics Laboratories in San Luis Obispo, CA. Total nitrogen and specific gravity were measured and analyzed per standard approved established protocols (Laboratory Test Directory, 2021). Participants were provided collection containers (Fisherbrand Low Form 24-h Urine Collection Container, Fisher Scientific) for 24-h total urine collection at the beginning of the study and were required to keep the samples refrigerated until delivered for analysis.

*NB* was determined using the standard nitrogen balance equation below [13], with other miscellaneous nitrogen excretion assumed to be 4 g/day [14].

$$NB=Nitrogen\;intake\;\left(g\right)-\left[Urinary\;urea\;nitrogen\;\left(g\right)+4\right]$$

NB=Nitrogen In−Nitrogen Out

### 2.7. Statistics

JMP Pro (Version 14.0) (SAS Institute, Cary, NY, USA) was used for all calculations for the study. Paired sample *t*-tests were used to assess differences between pre- and post-protein supplementation for significant physiological change (body composition) and urinary urea nitrogen change, both overall and per supplementation group. Independent *t*-tests were utilized to assess significant differences between the almond and whey groups. The paired sample *t*-test was utilized to assess significance between groups. More precisely defined, it is used to determine whether the mean difference between two sets of observations is zero. Normality was assessed utilizing the Shapiro–Wilks test before conducting analyses. Data was considered normal at the *p* = 0.05 level. In the case of the paired sample *t*-test, data passed normality tests if the differences between sample subsets were determined to be normal. Through the outlined analyses, all data passed normality tests and no transformations were needed. Observations were grouped by protein assignment type and then assessed for significance due to protein source. Descriptive statistics were utilized to quantify the mean and standard deviation of height, weight, and age. Calculations regarding nutrient intake included mean calorie intake, protein, carbohydrates, and lipids, as well as the percent intake of each macronutrient.

## 3. Results

### 3.1. Baseline Characteristics

An online screening questionnaire applying inclusion/exclusion criteria for study participation was completed by 192 individuals. Of these, 45 individuals were deemed eligible for inclusion in the study, and 20 participants followed through, enrolled, and successfully completed the study. Eligible participants were females aged 20–25 years, with no underlying health conditions, who did not regularly receive protein supplements (3× or more/week), were not pregnant, and did not receive any medication. Baseline characteristics of these individuals included: age of 21.9 ± 1.21 years and body weight of 60.85 ± 7.45 kg. Average BMI was 22.29, placing all participants in the “normal” classification or “standard” body category.

### 3.2. Body Composition

Baseline body fat was measured via BIA to be 21.56 ± 5.89%, whereas DXA indicated a total body fat percentage of 30.30 ± 5.05%. Both methods were used to ensure that all participants were similar in terms of body composition at baseline. The disparity between the two methods of assessment recorded was determined to be statistically significant (*p* = 0.0021). While there is no universally accepted official standard for appropriate levels of body fat, the two commonly utilized references are the American Council on Exercise (ACE) and Beth Israel Winchester Hospital [15,16]. ACE suggests a range of 21–24% as “fitness” for women, without considering age, whereas Beth Israel Winchester Hospital suggests the ideal range for women 20–29 years old to be 21–32%. Hence, regardless of scale, our study participants demonstrated low levels of body fat when assessed via BIA but normal levels when assessed by DXA [15,16,17]. Additionally, the BIA assessment of body water percentage was determined to be in range, indicating adequate hydration. According to BIA analytical software, visceral fat (visceral fat index) was low, while the calculated BMR was determined to be average (Table 2) [17].

### 3.3. Nutritional Analysis

Analysis of participants’ diet records conducted via ESHA determined an average caloric intake of 1672.7 ± 492.36 kcal/day. Typical dietary caloric intake recommendations include 2000 kcal/day for females [18]. In this context, our findings represent an under-consumption of calories by 328 kcal/day in the observed population. However, the percentages of kcal from each macronutrient were within the recommended range for all three macronutrients (carbohydrates, lipids, protein), with 48% of daily calories from carbohydrates (201.66 g), 36.2% from lipids (68.41 g), and 15.7% from protein (65.70 g). These percentages for carbohydrates and protein are in accordance with the acceptable macronutrient distribution ranges (AMDR) of 45–65% for carbohydrates and 10–35% for protein, according to the Institute of Medicine (now called: The National Academy of Medicine) [19,20].

The AMDR for lipids is 20–35%; hence, our participants demonstrated a slight over-consumption of lipids in terms of daily energy contribution. This percentage represents total lipids and does not consider the source and/or type (i.e., plant vs. animal and saturated vs. unsaturated/polyunsaturated/monounsaturated). Additionally, participants performed cardio workouts more frequently than strength workouts and for longer durations. The average time for a cardio workout varied greatly, between 0 to 120 min per day, yielding an average of 33.1 ± 36.4 min, whereas strength workouts were found to last 8.5 ± 19.6 min, on average. Most cardio workouts consisted of low-impact workouts, such as walks around town or to various buildings on campus. This indicates that participants are “lightly active”, on average, categorized as activity including daily living, plus 30–60 min per day of moderate activity, such as walking [20].

### 3.4. Effects of Protein Supplementation on Body Composition

All body composition values for pre- and post-supplementation comparisons are as indicated via BIA. Pre-supplementation values include all participants and are not separated by assigned protein supplement (Table 2). After completion of protein supplementation, participant weight increased by an average of 0.785 kg (*p* = 0.0047), representing a significant weight change during the supplementation period (Table 2). No significant weight change difference was observed between the almond and whey protein supplemented groups (*p* = 0.63) (Table 3). Changes in body fat percentage were not determined to be significant overall (*p* = 0.59) (Table 2); however, changes in body fat % between the whey and almond groups post-supplementation were determined to be significant when assessing the body fat % status at the end of the supplementation period between the two groups (*p* = 0.033) (Table 3). Comparison of pre- to post-supplementation for body fat % did not show a significant difference (when evaluating the change in body fat % supplementation conferred) regardless of supplementation type (almond or whey), and both groups exhibited a similar trend of a slight increase in body fat percent (Table 3). For the entire study population, muscle mass as a percentage of total weight remained statistically unchanged, regardless of protein supplementation source (Table 2). Thus, protein source does not seem to affect muscle for the time period and at the level of supplementation we introduced (8 days and 30 g/day of protein supplemented, respectively). Overall, no significant changes due to intervention were observed regarding body composition, and results are summarized in Table 2 and Table 3.

### 3.5. Protein Supplementation Effects on Urine Specific Gravity and Body Water %

Urine-specific gravity (USG) was assessed by calculating the ratio of urine density to the density of pure water [21]. USG was determined to be 1.01 ± 0.0067, placing participants within the normal/physiological range of 1.005 to 1.030 [22]. Following protein supplementation, USG increased slightly (1.011 ± 0.00655), regardless of protein source. This change was not determined to be significant (*p* = 0.26), and it can be concluded that increased protein intake did not impact hydration levels significantly, as also confirmed by body water % measurements via BIA (Table 2 and Table 3).

### 3.6. Protein Supplementation Effects on Urine Urea Nitrogen

In our study, all 20 participants exhibited low urine urea nitrogen (UUN) prior to supplementation (5.89 ± 2.20 g/24 h), placing them well below the typical range of 12–20 g/24 h. After supplementation, UUN levels increased significantly overall to 7.80 ± 1.91 g/24 h (*p* = 0.0016) (Table 4). In terms of nitrogen balance, on average, participants were determined to be in a very slight state of positive nitrogen balance overall (0.585 g/24 h), representing a state of anabolism and a sufficient nitrogen pool (Table 4). However, almost half (n = 9) of participants were in a negative nitrogen balance, indicative of a low protein intake [23].

Nitrogen balance (NB) was also increased significantly following supplementation, from 0.585 g of nitrogen to that of 3.660 g of nitrogen (*p* < 0.0001). The protein source effect was not determined to be significant (*p* = 0.7760) when comparing the magnitude of change in nitrogen balance, thus indicating that almond protein powder functions similarly to whey in this sense. A nitrogen balance from between −4 to −5 g/day to +4 or +5 g/day is typically considered equilibrium, thus indicating that overall, our participants were in a slightly anabolic state prior to supplementation, and protein supplementation furthered that anabolic state. Table 5 indicates nitrogen intake (NI) for the almond and whey groups, as assessed via dietary records. The difference in N-balance (change conferred) was not significantly different between the almond and whey (Table 5). No significant difference was found between protein sources in terms of nitrogen intake (*p* = 0.9494), thus indicating that the amounts of both the almond and whey protein extended similar effects on nitrogen status in the body (Table 5).

## 4. Discussion

### 4.1. Body Composition

Both DXA and BIA are frequently used to assess body composition; however, the results indicated a significant difference between the two methods in terms of amounts of the components of body composition, including body fat. Our observations on the differences in body fat assessment between BIA and DXA closely align with previous research reports [24,25,26], observing BIA to consistently produce a lower body fat percentage compared to DXA, particularly in females, which, as per sex, is reasonable since females exhibit a higher body fat percentage under normal body composition compared to their male counterparts. The differences observed between the two assessment methods (i.e., BIA vs. DXA) may be attributed to the measurement method used (DXA versus BIA) and the formulae utilized by the built-in software of the equipment to calculate body fat. Different equations used to predict/extrapolate body fat can result in large variations between DXA and BIA, ranging from 0.3–8.1% body fat [27]. We utilized DXA at baseline to compare our participants relative to the extensive DXA-derived databases and ensure those who participated had values within the normal range. BIA was used at baseline and post-intervention to assess the effects of protein supplementation on body composition.

### 4.2. Nutritional Analysis

Fat source is not considered in the AMDR, nor was it considered in this study. In the case of our study, participants frequently consumed avocados and peanut butter and rarely consumed sweets and red meat, according to their dietary records. It is, therefore, plausible that the relatively high fat-derived energy intake percentage is primarily due to the unsaturated fat consumed, as this was also observed by others [28,29]. Nutritional analysis indicated adequate intakes and the reasonably aligned contribution of each macronutrient, revealing desirable dietary habits in this regard. It is important to note that all participants had ensured basic nutrition knowledge (an introductory nutrition college-level class had been taken), and the observed eating habits may not have been representative of those of the campus’ general student population. The observed body composition and dietary habits were aligned with research conducted by Hong et al., whereby the BMI of nutrition majors was compared to that of non-nutrition majors. A total of 202 female students were assessed, concluding that nutrition majors exhibited a significantly lower BMI than that of non-nutrition majors [30]. The biggest factor in this finding was deemed to be nutrition knowledge informing food choice; nutrition majors were more likely to choose a healthier meal option, whereas non-nutrition majors were more likely to choose a meal based on convenience [30]. Due to the characteristics of our studied population, it is likely that a higher-than-average nutrition knowledge, coupled with possibly more interest and motivation towards nutrition and health, may at least partially explain the dietary habits of our study participants. It seems plausible that their knowledge and interest in nutrition is an advantage in this type of study as it may improve compliance and adherence to the consistent pattern of intake requested.

### 4.3. Effects of Protein Supplementation on Body Composition

Weight change among the participants of the observed population during the intervention was 0.8 kg or a 1.3% increase. This observed weight change may be attributed to the increased caloric intake due to the protein shakes and can be anticipated. Participants were asked to continue their dietary intake, as noted in their food records, while adding the protein shakes daily. Hence, the additional daily calories of 570 kcal for the almond protein shake and 410 kcal for the whey protein shake likely contributed to weight gain. It is important to note that while this change was determined to be significant, it is a relatively small change overall. Had this study been longer and weight change continued to increase, this change would be a more concerning finding. However, weight gain over a longer period of protein supplementation and an ad libitum food intake may not be problematic due to the satiating effects of protein [31], and it is likely that longer supplementation periods would show little to no weight gain or, possibly, weight loss.

In terms of body fat %, the magnitude of change was similar for both types of supplementation (i.e., almond and whey), even though the post-supplementation body fat % status for almond was higher. Notably, however, our focus was to assess the change in difference (Δ; delta) in nitrogen balance, not necessarily optimizing body composition. We thus prioritized nitrogen equalizing rather than calorie equalizing.

### 4.4. Protein Supplementation Effects on Urine Specific Gravity

The lack of significant difference between USG levels pre- and post-supplementation is of interest due to current dietary trends, which often include high protein diets. High protein diets are often recommended due to protein’s satiety effects and its capacity to increase lean muscle mass when appropriately combined with exercise [32]. However, such practice, if not regulated, may lead to excess protein intake and concerns regarding dehydration [33]. Our protein supplementation did not appear to negatively impact hydration status.

### 4.5. Protein Supplementation Effects on Nitrogen Status

It should be noted that consistent exercise has been shown to increase protein requirements to levels above the RDA of 0.8 g/kg/day. The degree to which protein requirements increase is variable and dependent upon several factors, but significant evidence has suggested that adequate dietary protein to meet demands and positively influence the effects of exercise is likely between 1.2–2.0 g/kg/day, as also stated in the official position of the Academy of Nutrition and Dietetics of Canada and the American College of Sports Medicine as well as the International Society of Sports Nutrition [34,35]. More specifically, exercise suggests endurance exercise, warranting approximately 1.2–1.4 g/kg/day, while higher intensity resistance-training requirements may well be 1.6–2.0 g/kg/day [36]. In the current study, the average protein intake for all participants at baseline was approximately 1.08 g/kg/day (see NI of Table 4; BW of Table 3). At that protein level, 9 of the 20 participants were in negative nitrogen balance, and this could be due to the demands of each participant’s exercise regimen. The protein supplementation of 30 g/day increased the average protein intake to approximately 1.55 g/kg/day (see NI of Table 4; BW of Table 3) and significantly increased the average nitrogen balance value. These data suggest that in this cohort, with the reported exercise activity and calorie intake, the protein requirement could be more plausibly between 1.08–1.55 g/kg/day and that protein supplementation may be effective in providing positive health effects.

Protein supplementation further enhanced positive nitrogen balance, regardless of protein source. While nitrogen balance is prone to bias, it produced similar results in terms of the direction in both groups, increasing NB. Such increase is of interest in several areas of health and nutrition. When combined with resistance exercise, positive NB aids in muscle growth and MPS. This is due to the increased amino acid pool associated with increased nitrogen intake. Consumption of EAAs immediately following exercise has been shown to significantly increase MPS 24 h after resistance exercise when compared to rest [10]. Protein consumption immediately following exercise has been linked to increased MPS on several occasions [37]. This is due to the increased amino acid concentration in the blood, combined with the stimulation of muscle tissue following resistance exercise. However, popular plant-based protein supplements, such as soy and pea protein, have sometimes displayed decreased effects on MPS in comparison to whey, although results appear inconsistent [9,10]. Supplementation with whey and almond protein following resistance training would be of interest to assess the protein source impact on MPS, in addition to NB, to further investigate the effect of plant-based proteins and muscle growth, also taking into consideration different protein profiles and quality as per amino acid profiles due to different plant sources.

In the present study, 30 g of whey or almond protein appeared to provide similar effects in terms of NB, despite the different amino acid contents/profiles. This approach of supplementation with almond-based protein may be beneficial for several reasons, including the desire to increase protein in a vegan diet, the desire to increase muscle mass without consuming whey protein (animal products), or the desire to consume a higher protein diet. Higher protein diets have become popular due to their satiety effects, which may aid in weight loss. Between 2013 and 2014 alone, there was a 49% increase in snacks making high protein claims [38], further illustrating this trend. Protein intake is believed to stimulate metabolic hormones that communicate energy status to the brain in a process termed signaling fullness.

Due to the novelty of almond protein powder, there are considerable research opportunities to further explore the functionality of this protein powder. Potential disadvantages of almond protein include allergic reactions and, perhaps, less satiety relative to whey. Moreover, there is a difference in the absorption and metabolism rate between whey and almond, favoring the former. This may create a difference in the rate of muscle building over time. One interesting insight would, thus, be a longer supplementation period to observe changes to muscle mass and body fat. Four to six weeks are regarded as the standard for muscle mass change. Adherence to the protein shake consumption protocol for this time period would help to determine if, over time, almond protein affects muscle mass, in addition to UUN and NB levels. Furthermore, since certain amino acid profiles may be able to improve chronic conditions such as sarcopenia and Type 2 diabetes management [39], it would be interesting to investigate how plant protein powders could be utilized in this regard. Additionally, the investigation of ways to improve, enhance, and optimize the amino acid profile and the quality/value of almond and, potentially, other plant-based proteins would be of significant interest and encouraging, given our results presented herein. Limitations of this study include the limited length of intervention, the convenient mode of sample selection, and the inclusion of female participants only. It would be interesting in this regard to investigate the same outcome variables, including body composition, for a longer supplementation period, on both sexes, and also in varying age groups.

## 5. Conclusions

The overall objective of the research presented herein was to assess the functionality of almond protein on NB in comparison to whey protein. It was hypothesized that the two proteins would increase NB significantly and similarly due to the increased protein in-take. No significant difference was observed between protein sources on NB, and NB was increased overall from 0.585 g to 3.66 g. This indicates that almond protein powder may be a good vegan substitute for whey protein powder. This is particularly important considering the popularity of almond products in the United States, especially in the non-dairy milk alternative market. Several other plant-based options that may be more sustainable exist; however, the growing popularity of almonds and almond milk place this nut in a position to possibly lead the shift in dietary habits and increase sustainable diets in the United States. The observed functionality of almond protein powder addresses the myth that plant-based proteins do not provide adequate protein, while it highlights a beneficial product that may encourage more consumers to follow a plant-based diet.

## Figures and Tables

**Table 1:** Ingredient breakdown of each shake. Each shake totals 15 g of protein for each of the daily servings.

Ingredient per Drink	Almond Shake (Plant Source)	Whey Shake (Animal Source)
Unsweetened Almond Milk (mL)	295	295
Chocolate Flavoring (g)	16.8	16.8
Protein Powder * (g)	34.5	17

* Amount of protein powder required to provide 15 g of protein.

**Table 2:** Body composition characteristics of female college students, pre- and post-supplementation. Statistical significance was determined via matched paired *t*-test and is on a per-row basis. BMR: basal metabolic rate calculated using BIA equipment software. Values are means ± SD.

Measure	Pre-Supplementation (Baseline)	Post-Supplementation (Overall)
Body weight (kg)	60.84 ± 7.67 ^a^	61.63 ± 8.16 ^b^
Body fat (%)	21.5 ± 6.03 ^a^	23.29 ± 5.30 ^a^
Muscle mass (%)	33.75 ± 2.58 ^a^	33.01 ± 2.28 ^a^
Body water (%)	51.97 ± 3.83 ^a^	51.15 ± 4.01 ^a^
Visceral Fat Index	1.25 ± 0.64 ^a^	1.40 ± 0.75 ^a^
BMR	1446.65 ± 136.06 ^a^	1441.00 ± 134.74 ^a^

Different letters in superscript denote statistically significantly different at *p* < 0.05.

**Table 3:** Body composition characteristics of female college students in each supplementation group. Statistical significance was determined via matched paired *t*-test and is on a per-row basis. BMR: basal metabolic rate calculated using BIA equipment software. Values are means ± SD.

Measure	Pre-Supplementation (Almond)	Pre-Supplementation (Whey)	Post-Supplementation (Almond)	Post-Supplementation (Whey)
Body weight (kg)	61.23 ± 8.71 ^a^	60.45 ± 6.49 ^a^	62.14 ± 9.65 ^a^	61.11 ± 6.85 ^a^
Body fat (%)	22.62 ± 6.00 ^a^	20.38 ± 6.16 ^a^	24.42 ± 5.06 ^b^	22.15 ± 5.56 ^a^
Muscle mass (%)	33.27 ± 2.58 ^a^	34.24 ± 2.63 ^a^	32.51 ± 2.15 ^a^	33.5 ± 2.41 ^a^
Body water (%)	52.46 ± 4.41 ^a^	51.48 ± 3.33 ^a^	51.58 ± 4.95 ^a^	50.71 ± 3.00 ^a^
Visceral Fat Index	1.30 ± 0.67 ^a^	1.20 ± 0.63 ^a^	1.40 ± 0.84 ^a^	1.40 ± 0.70 ^a^
BMR	1435.00 ± 155.26 ^a^	1458.30 ± 121.10 ^a^	1434.40 ± 159.08 ^a^	1447.60 ± 113.42 ^a^

Different letters in superscript denote statistically significantly different at *p* < 0.05.

**Table 4:** Summary of nitrogen balance changes pre- and post-supplementation, not separated by protein source (see materials and methods section for the method of NB calculation/formula; + indicates positive nitrogen balance). NI: nitrogen intake, UUN: urinary urea nitrogen (nitrogen out). Values are means ± SD; *t*-test applied on a per-row basis.

Measure	Pre- Supplementation (Baseline)	Post- Supplementation (Overall)
NI (g)	10.48 ± 3.80 ^a^	15.28 ± 3.80 ^a^
UUN (g)	5.89 ± 2.20 ^a^	7.80 ± 1.91 ^b^
N-balance (g)	+0.585 ± 4.59 ^a^	+3.660 ± 5.20 ^b^

Different letters in superscript denote statistically significantly different at *p* < 0.05.

**Table 5:** Summary of nitrogen balance changes pre- and post- supplementation, including protein source. Statistical significance was determined using matched paired *t*-tests and is on a per-row basis (see materials and methods section for the method of NB calculation/formula; + indicates positive nitrogen balance). NI: nitrogen intake, UUN: urinary urea nitrogen (nitrogen out). Values are means ± SD.

Measure	Pre- Supplementation (Almond)	Pre- Supplementation (Whey)	Post- Supplementation (Almond)	Post- Supplementation (Whey)
NI (g)	9.62 ± 3.99 ^a^	11.33 ± 3.60 ^a^	14.42 ± 3.99 ^a^	16.14 ± 3.60 ^a^
UUN (g)	6.31 ± 2.81 ^a^	5.47 ± 1.39 ^b^	8.25 ± 3.63 ^b^	7.35 ± 2.51 ^b^
N-balance (g)	−0.69 ± 4.96 ^a^	+1.86 ± 4.02 ^b^	+2.53 ± 6.10 ^b^	+4.79 ± 3.83 ^b^

Different letters in superscript denote statistically significantly different at *p* < 0.05.

## Data Availability

Data are available upon request from corresponding author. Furthermore, data are reported in Adeline Maykish’s Master of Science thesis, available publicly through the Digital Commons @ Cal Poly system upon release, and the Robert E. Kennedy Library of California Polytechnic State University.
